# Citrulline uptake in rat cerebral cortex slices: Modulation by Thioacetamide -Induced hepatic failure

**DOI:** 10.1007/s11011-013-9472-5

**Published:** 2014-01-03

**Authors:** Magdalena Zielińska, Marta Obara-Michlewska, Wojciech Hilgier, Jan Albrecht

**Affiliations:** Department of Neurotoxicology, Medical Research Centre, Polish Academy of Sciences, Pawinskiego 5, 02-106 Warsaw, Poland

**Keywords:** Acute liver failure, Thioacetamide, Citrulline, Argininosuccinate syntethase, Argininosuccinate lyase, Nitric oxide

## Abstract

L-citrulline (Cit) is a co-product of NO synthesis and a direct L-arginine (Arg) precursor for de novo NO synthesis. Acute liver failure (ALF) is associated with increased nitric oxide (NO) and cyclic GMP (cGMP) synthesis in the brain, indirectly implicating a role for active transport of Cit. In the present study we characterized [^3^H]Cit uptake to the cortical brain slices obtained from control rats and rats with thioacetamide (TAA)-induced ALF (“TAA slices”). In both control and TAA slices the uptake was partially Na^+^-dependent and markedly inhibited by substrates of systems L and N, including L-glutamine (Gln), which accumulates in excess in brain during ALF. Cit uptake was not affected by Arg, the y^+^/y^+^L transport system substrate, nor by amino acids taken up by systems A, x_c_^−^or X_AG_. The V_max_ of the uptake in TAA slices was ~60 % higher than in control slices. Chromatographic (HPLC) analysis revealed a ~30 % increase of Cit concentration in the cerebral cortical homogenates of TAA rats. The activity of argininosuccinate synthase (ASS) and argininosuccinate lyase (ASL), the two enzymes of Cit-NO cycle catalyzing synthesis of Arg, showed an increase in TAA rats, consistent with increased ASS and ASL protein expression, by ~30 and ~20 %, respectively. The increased Cit-NO cycle activity was paralleled by increased expression of mRNA coding for inducible nitric oxide synthase (iNOS). Taken together, the results suggest a role for Cit in the activation of cerebral NO synthesis during ALF.

## Introduction

**Scheme 1:**
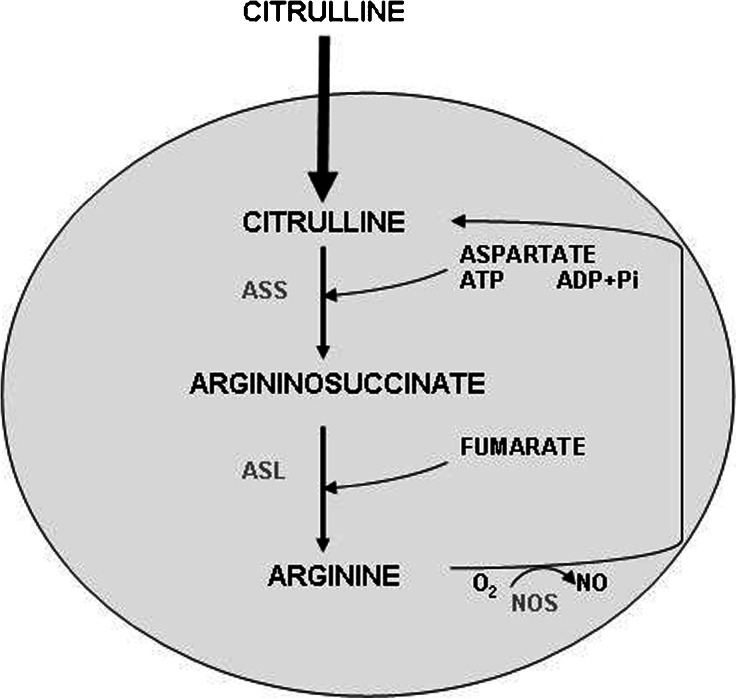
The Cit-NO cycle in the brain. As suggested by this study, in TAA-induced ALF, increased amounts of Cit enter the brain cells, due to increased Cit uptake activity (as indicated by *bold arrow*). The increased Cit content in the brain tissue, stimulation of ASS and ASL, and increased iNOS expression, cooperatively enhance the Cit-NO cycle activity, positively modulating the NO tissue content

Hepatic encephalopathy (HE), a consequence of acute or chronic liver failure (ALF or CLF), is a complex neuropsychiatric disorder that results from impaired clearance from blood of ammonia and other toxins, and is compounded by peripheral or local inflammatory processes (Prakash and Mullen 2010). The cellular and molecular mechanisms underlying HE are complex, but are as a rule associated with interference of ammonia with various aspects of brain metabolism, leading to imbalance of neural transmission (Albrecht and Jones 1999; Felipo and Butterworth 2002). At the molecular level the activation of ionotropic (mainly NMDA) glutamate receptors leads to increased intracellular free calcium which, after binding to calmodulin, activates nitric oxide synthase (NOS), leading to increased production of nitric oxide (NO) (Garthwaite et al. 1988). In ALF, ammonia-induced increase of NO and subsequently extracellular cGMP is a good indicator of the over-stimulation of NMDA receptors in rat brain, a process that contributes to increased reactive oxygen and nitrogen species (ROS/RNS) production (Kosenko et al. 2003; Hermenegildo et al. 2000; Hilgier et al. 2004). Increased NO synthesis under HE conditions in the brain, requires L-arginine (Arg) as a substrate for NOS, which generates NO and L-citrulline (Cit). Arg is a semi-essential amino acid in CNS and its availability depends both upon its uptake from the circulation (Fotiadis et al., 2013) and the recycling of Cit to Arg in Cit-NO cycle (Zhang et al. 1999, see also Scheme 1). Cit generated as a by-product of NO synthesis can be recycled to Arg in reactions subsequently catalyzed by argininosuccinate synthetase (ASS; EC 6.3.4.5) and argininosuccinate lyase (ASL; EC 4.3.2.1) via the Cit-NO cycle (Scheme 1). The activity of ASS and ASL was found increased in cerebral cortex of hyperammonemic rats (Swamy et al. 2005). Also, increased Arg uptake has been repeatedly demonstrated in ammonia-exposed synaptosomes (Westergaard et al. 1993; Rao and Butterworth 1996; Rao et al. 1997; Rao 2002) and astrocytes (Hazell and Norenberg 1998). Infusion of ammonium acetate to rats increased serum level of Arg, suggesting increased availability of Arg for NO synthesis (Ishihara et al. 1998). However, in TAA-induced ALF, the total blood to brain barrier transport of Arg was decreased, as determined by the brain uptake index (BUI) (Albrecht et al. 1996). Moreover, TAA-induced HE was associated with increased conversion of Arg to neurotransmitters Glu and γ-aminobutyric acid (GABA), a process engaging increased activities of arginase (AR; EC 3.5.3.1) and ornithine aminotransferase (OAT; EC 2.6.1.13) (Albrecht et al. 1990). On the other hand, elevated concentration of L-glutamine (Gln) in hyperammonemic rat brain inhibits cGMP synthesis by interaction with Arg transport into the cells (Zielinska et al. 2011), which could counter the increase of NO synthesis by ammonia. One other mechanism by which Gln could affect NO synthesis in the brain is by interference with Cit recycling to Arg, as shown by Wu and Meininger (1993) in peripheral endothelial cells. The potential importance of Cit recycling for Arg availability and NO synthesis in HE affected brain prompted us to analyze the as yet not considered Cit transport as a critical step within the Cit-NO cycle. The present study is, to the best of our knowledge, the first which attempted to characterize in more detail Cit transport in ex vivo brain tissue.

## Experimental procedures

### HE model

Adult male Sprague–Dawley rats (150–180 g) were reared under standard conditions at the local animal facility. The animals had free access to food and water. All experiments were performed with agreement of local animal ethical committee that approved the experimental design. HE with cerebral metabolic changes and symptoms typical of acute HE was induced by 3 i.p. injections of thioacetamide (TAA) (250 mg per kg of body weight) at 24 h hours intervals (Hilgier and Olson 1994; Hilgier et al. 1996) and sacrificed 24 h after third injection. Control rats were analogically injected with sodium saline solution.

### Cerebral cortical slices

Male Sprague-Dowley rats (150–180 g) were used throughout. In essence, a previously described procedure was followed (Zielińska et al. 1999) with slight modifications. Animals were decapitated and the brains were immediately transferred into ice-cold Krebs-Ringer bicarbonate buffer (Krebs buffer) of the following composition: 118 mM NaCl, 25 mM NaHCO_3_, 4.7 mM KCl, 1.2 mM KH_2_PO_4_, 2.5 mM CaCl_2_, 1.2 mM MgSO_4_, 10 mM glucose, aerated with 95 % O_2_ and 5 % CO_2_ at pH 7.4. After preparation the cortices were cut into 300 μm slices using a manual chopper. The slices were transferred to borosilicate glass vials containing Krebs buffer. Each vial received its own supply of a 95 % O_2_ and 5 % CO_2_ gas mixture. The slices were pre-incubated under these conditions for 30 min in a water bath at 37.4 °C.

### Uptake experiments

After pre-incubation of cerebral cortical slices the uptake was started by adding [^3^H]Cit at 100 μmol/L final concentration and the incubation was continued for 7 min. Kinetics of [^3^H]Cit uptake was determined in Na^+^-containing medium over varying extracellular Cit concentrations (2.5–1,000 μM). Substrate preference analysis was measured in a competition study where 100 μM/L [^3^H]Cit was used together with unlabelled Cit in the presence of a number of 0.5–10 mM competing unlabelled L-amino acids or MeAIB (all substrates were from Sigma- Aldrich, USA).

The incubation was terminated by a rapid vacuum filtration through 2.5 cm 0.45 μm Millipore filter disks (Millipore, Ireland), followed by three washes with 2 ml with Krebs buffer maintained at 4 °C. The radioactivity on filter disks was measured in a Wallac 1409 Liquid Scintillation Counter (Perkin - Elmer, Finland). Correction for the remaining citrulline in the extracellular space was included in blank samples, defined as zero-time incubation (<20 s) and accounted for about ~ 7–10 % of the total uptake. Protein determination was performed according to Bradford’s procedure (Bradford 1976).

### Argininosuccinate synthetase and argininosuccinate lyase activity measurements

Argininosuccinate synthetase (ASS) and arginosuccinate lyase (ASL) activity was measured by a modification of a method of Swamy et al. (2005). Briefly, the cortical tissue was weighed and homogenized in 0.05 M phosphate buffer at pH 7.3 and 4 °C. The assay of ASS was started by the addition of 0.2 ml of 20 % homogenate to the reaction mixture contained Cit, aspartic acid, ATP, magnesium chloride, each at 0.01 M concentration and 21U of arginase. The reaction was conducted at 37 °C and stopped after 1 h by addition of 0.2 ml of 50 % trichloracetic acid. The reaction mixture was then centrifuged and supernatant was used for colorimetric determination of urea. The absorbance was read at 540 nm. The reaction mixture for ASL assay contained argininosuccinate at 6.0 mM and 10.5 U of arginase and was started by addition of 0.2 ml of 20 % homogenate to the reaction mixture.

### L-citrulline determination in cerebral cortical homogenates

Cit was analysed using HPLC with fluorescence detection after derivatisation in a timed reaction with o-phthalaldehyde plus mercaptoethanol, as described earlier (Zielińska et al. 1999). Derivatised samples (50 μl of microdialysate) were injected onto 150 × 4.6 mm 5 μm Hypersil ODS column, eluted with a mobile phase of 0.075 M KH_2_PO_4_ solution containing 10 % (v/v) methanol, pH 6.2 (solvent A), and methanol (solvent B). The methanol gradient was 20–70 % and the elution time 20 min.

### Protein isolation and Western blot analysis

Isolated rat brain cerebral cortexes were homogenized and centrifuged at 4 ºC with Triton Lysis Buffer as described earlier (Zielinska et al. 2011). Total protein concentration in supernatants was determined by the Lowry method using Modified Lowry Protein Assay Reagent (Pierce). Protein (30 μg) was mixed with sample loading buffer, separated on SDS-PAGE and then transferred onto nitrocellulose membrane. The membranes were blocked with 5 % non-fat dry milk in TBS-T buffer. Incubation with antibodies against ASS (1:1,000, Sigma-Aldrich, USA) and ASL (1:1,000, Sigma-Aldrich, USA) was done in TBS-T buffer with 5 % non-fat dry milk at 4 °C temperature over night followed by 10 min incubation with peroxidase-conjugated-anti-rabbit antibodies (1:2,500, Sigma-Aldrich, USA) for detection by SuperSignal West Pico Chemiluminescent Substrate (Pierce). The first antibody was stripped off with 0.1 M glycine, pH 2.9, and second incubation was performed with an antibody against GAPDH (1 h at room temperature), (1:5,000, Sigma-Aldrich, USA).

### Real-time PCR analysis

Total RNA was isolated using TRI Reagent (Sigma-Aldrich, USA), and then 1 μg was reverse-transcribed using the High Capacity cDNA Reverse Transcription Kit (Applied Biosystems, Life Technologies). Real time PCR was performed in 96 well plates with the ABI 7500 apparatus (Applied Biosystems, Life Technologies) using the MGB Taqman probe assay. Probes for iNOS and endogenous control β-actin were purchased from Applied Biosystems (Rn 00561646-m1 and Rn 00667869-m1, respectively). Each reaction contained 5 μl Taqman Universal PCR Mastermix in a total volume of 10 μl, and 1 μl cDNA was added to the reaction. The real time PCR reactions were performed at 95 °C for 10 min, followed by 40 cycles of 30 s at 95 °C and 1 min at 60 °C. The results of the analysis were calculated in relation to the β-actin product, and results were calculated according to, and expressed by an equation (**2** ^**-ΔΔCt**^ **)** that gives the amount of target, normalized to an endogenous reference and relative to a calibrator. C_T_ is the threshold cycle for target amplification (Livak and Schmittgen 2001).

### Statistical analysis

Statistical analysis of the data was performed using one-way analysis of variance followed by the Dunnet’s comparison test or the two-tailed Student’s test.

## Results

**Fig. 1:**
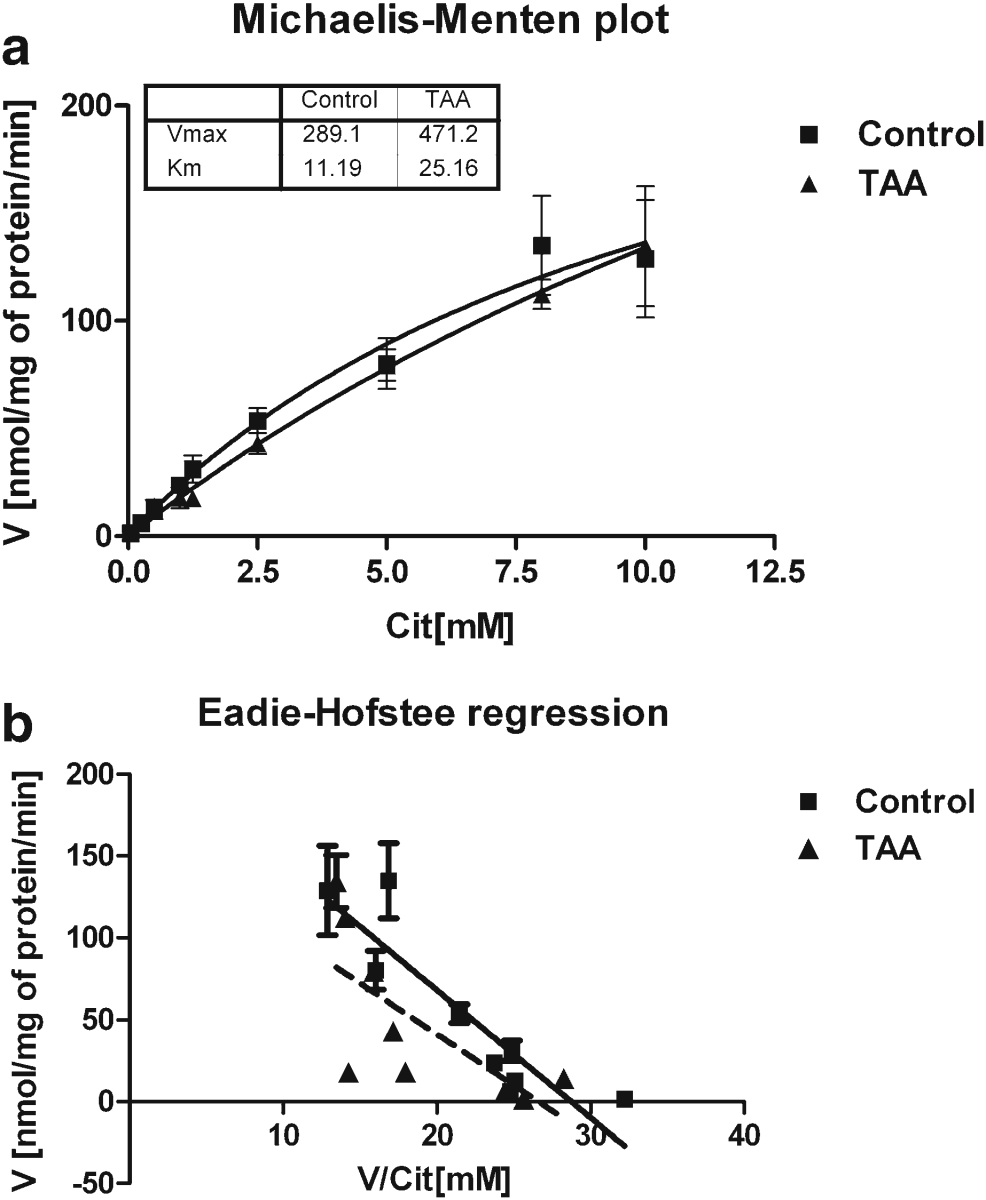
**a–b** Kinetics of L-citrulline transport measured in cerebral cortical slices derived from control and TAA rats and analyzed in three different modes. Results are mean ± SD (*n* = 4–5)

**Fig. 2:**
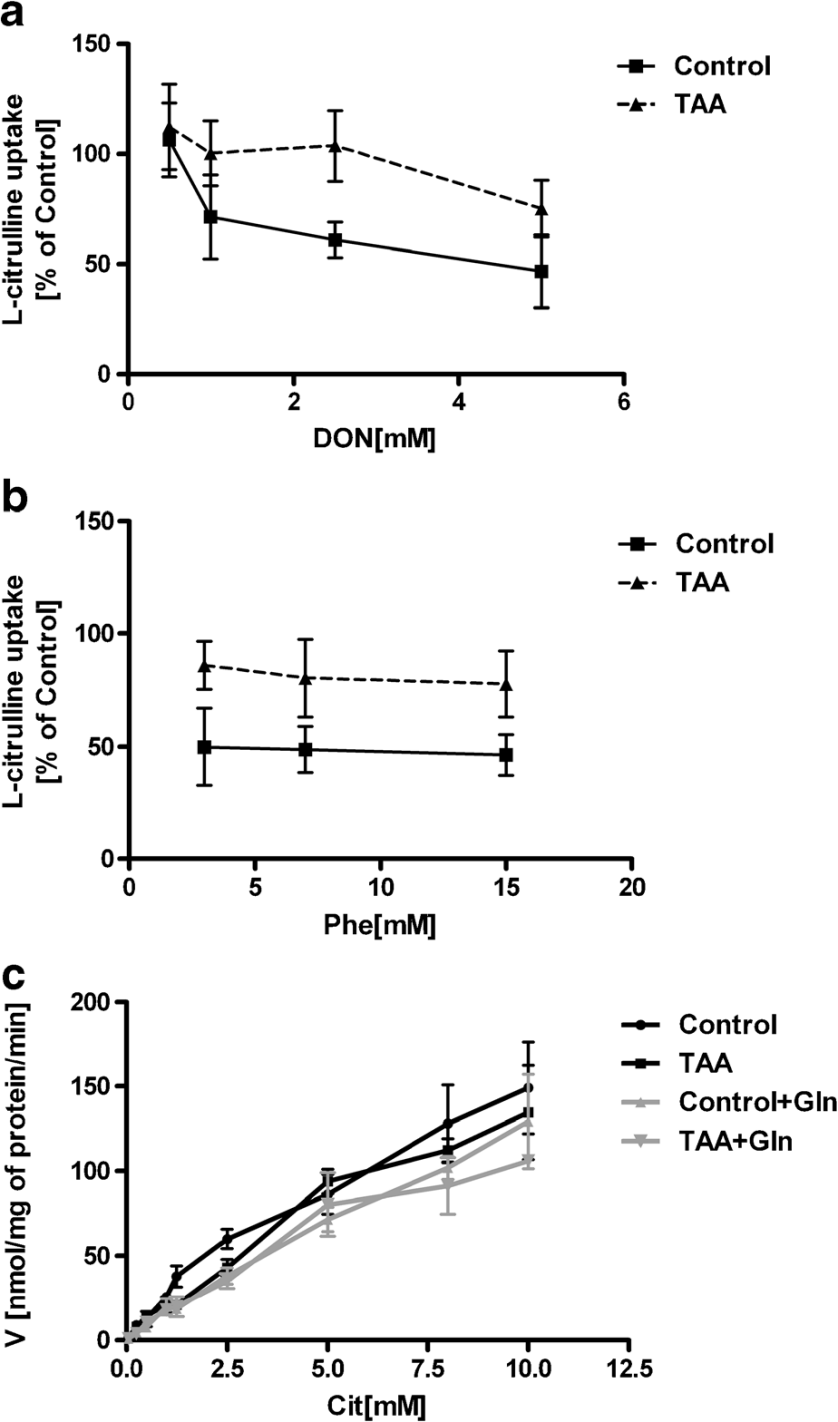
Specificity of L-citrulline transport in cerebral cortical slices from control and TAA rat brain. Inhibition of L-citrulline transport was measured in slices incubated with Na^+^ containing Krebs buffer with increasing concentrations (0.5–5 mM) of DON (A) or L-phenylalanine (B). (C) Kinetic analysis of Cit uptake in the presence of 5 mM Gln. Transport of L-citrulline (0.1 mM) was measured over 7 min, and expressed as a percentage of the transport rate determined in the absence of an inhibitor amino acid. Values are mean ± SD (*n* = 5–6)

Kinetics of [^3^H]Cit uptake to the rat cerebral cortical slices was analyzed using Michaelis–Menten nonlinear analysis (Fig. 1a) and linearized transformation Eadie–Hofstee (Fig. 1b). The analyzes revealed a simple, one component of the uptake, where V_MAX_ of increased from 289 ± 59 to 471 ± 84 pmol mg^−1^ protein x min^−1^, whereas K_M_, though slightly increased (from 11 ± 4 to 25 ± 6 mM) in slices obtained from TAA-induces ALF rats (TAA) (Fig. 1a). Transport of [^3^H]Cit was partially dependent on extracellular Na^+^, (Table 1) and significantly inhibited by a 10-fold excess of unlabelled Cit, L-histidine (His; substrate for system L), L-glutamine (Gln; substrate for systems L and N) or different concentrations of L-phenylalanine (Phe; substrate for system L) and 6-diazo-5-oxo-l-norleucine (DON; model substrate for system N). In contrast, 2-methylaminoisobutyric acid (MeAIB) and L-glutamate (Glu) (substrates for systems A and xc ^-^/X_AG_), respectively, were ineffective inhibitors, as well as L-arginine (Arg), a substrate for y^+^ system (Table 1). Detailed analysis of the inhibition of [^3^H]Cit transport in control and TAA slices by DON revealed inhibition in a concentration-dependent manner, reducing [^3^H]Cit uptake by ~54, ~39, ~29 % and ~70, ~55 and ~50 %, respectively at 5; 2.5; and 1 mM concentration (Fig. 2a). In contrast, the inhibition caused by 3 mM Phe was not further enhanced by increasing extracellular Phe to 15 mM (Fig. 2b).

**Table 1:** The effects of extracellular sodium and system-selective amino-acid substrates on L-citrulline uptake in cerebral cortical slices from control and TAA rats

Inhibitor	System selectivity	L-citrulline transport (% control)	
		Control	TAA
Na^+^	L,N,y^+^, x_c_, − X_AG_	103 ± 19	153 ± 18*
Na^+^- free	A, N	72 ± 20	89 ± 26**
L-arginine (10 mM)	y^+^	71 ± 29	101 ± 23
L-citrulline (10 mM)	?	58 ± 14*	82 ± 26**
BCH (10 mM)	L	48 ± 14*	62 ± 26**
L-glutamine (10 mM)	L,N	60 ± 14*	71 ± 23**
L-histidine (10 mM)	N	46 ± 15*	65 ± 21**
DON (5 mM)	N	47 ± 16*	75 ± 22**
MeAIB (10 mM)	A	98 ± 14	102 ± 26
L-glutamate	x_c_ − or X_AG_	90 ± 17	116 ± 20

Transport of L-citrulline (0.1 mM) was measured over 7 min in the absence or presence of Na^+^ ions and an excess of system-selective amino-acid substrates. Data are expressed as a percentage of the influx rate in control slices (100 % = 19.2 pmol mg protein-1 min-1). Values are mean ± SD of 4–6 experiments with three replicates in each experiment. *p < 0.05 vs. Control and **p < 0.05 vs. to TAA

**Fig. 3:**
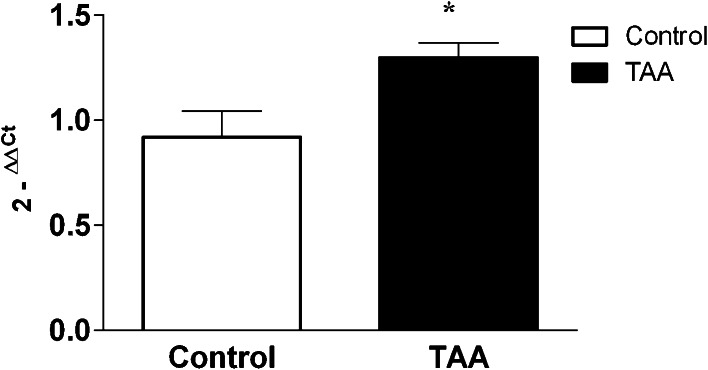
The expression of iNOS mRNA in the cerebral cortex of control and TAA rats. Results are mean ± SD; (*n* = 5) *p < 0.05 vs. “Control”

**Fig. 4:**
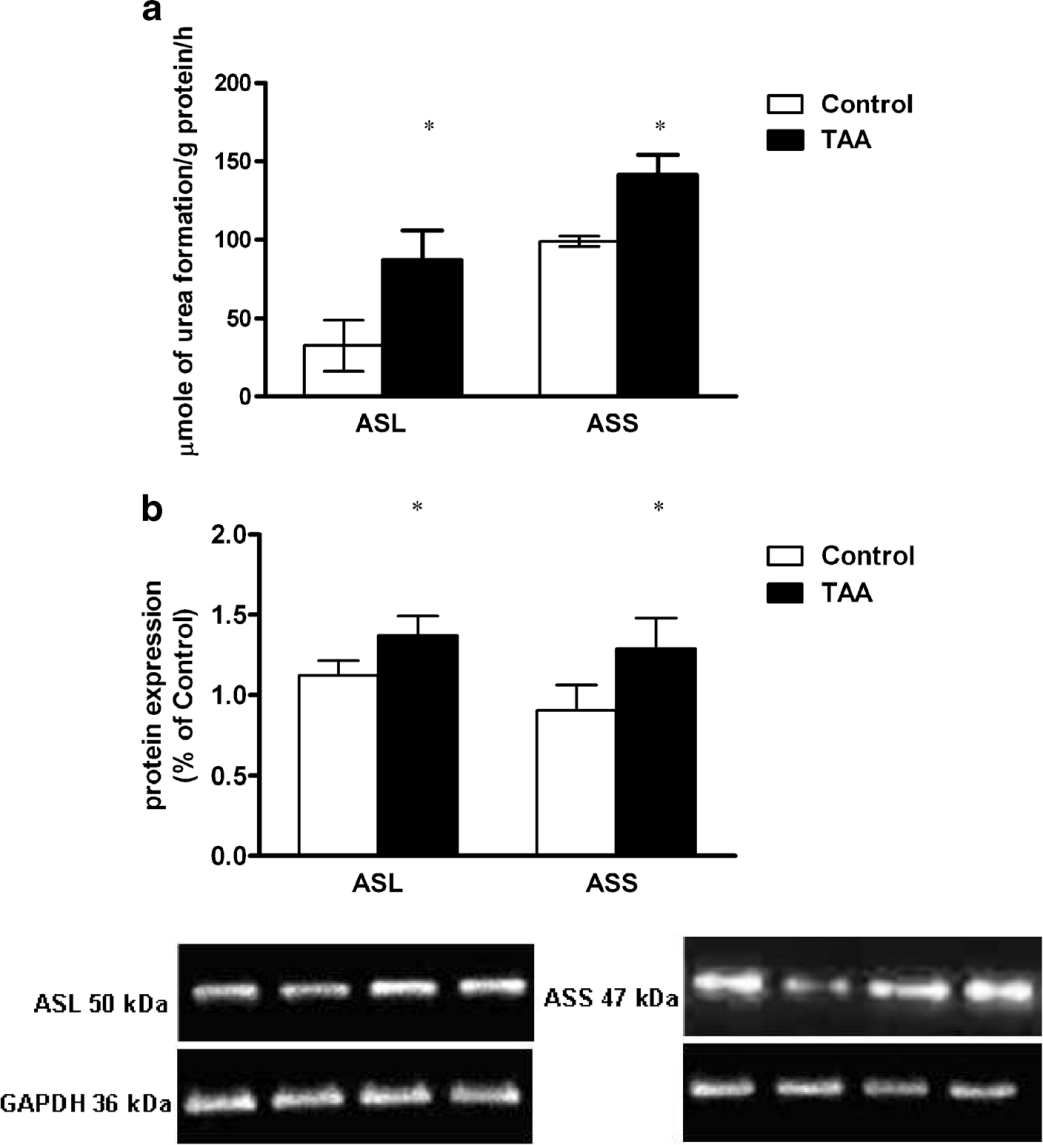
The activity (**a**) and protein expression (**b**) of arginosuccinate synthetase (ASS) and arginosuccinate lyase (ASL) in the cerebral cortex of control and TAA rats. Results are mean ± SD; (*n* = 4–5) *p < 0.05 vs. “Control”

Chromatographic analysis of cerebral homogenates obtained from control and TAA rats revealed an increase in total tissue concentration of Cit from 1.3 ± 0.2 to 1.7 ± 0.1 μM/g of tissue (Table 2). In TAA rats total tissue concentration of Arg increased from 10.6 ± 0.6 to 7.2 ± 0.2 (Table 2). The mRNA expression of iNOS was increased in TAA cortex by ~20 % of control, respectively (Fig. 3). TAA increased the activity of ASS (0.5-fold) and ASL (0.9-fold) in cerebral cortical homogenates (Fig. 4a). Western blot analysis showed increased expression of ASS (~0.3-fold) and ASL (~0.2-fold) in TAA rats (Fig. 4b).

**Table 2:** Total concentration of L-citrulline and L-arginine in cortical slices from control and TAA rats

	Amino acids concentration in rat brain cortex	
	(μmol/g wet tissue)	
	Control	TAA
L-citrulline	1.3 ± 0.2	1.7 ± 0.1*
L-arginine	10.6 ± 0.6	7.2 ± 0.2**

Results are mean ± SD; (*n* = 6) *p < 0.05 vs. “Control”

## Discussion

In the brain, astrocytes are the main site of ammonia detoxification, through the amidation of glutamate (Glu) forming Gln (Cooper and Plum 1987), in the reaction catalyzed by glutamine synthetase (GS) (Martinez-Hernandez et al. 1977). Accumulation of Gln in the brain in HE patients or animal models of ALF is one of the main factors that contribute to ammonia-induced neurotoxicity (Albrecht and Norenberg 2006). HE impairs the glutamate/NO/cGMP pathway, which includes interference with the synthesis of NO (Hermenegildo et al. 1998; 2000; Hilgier et al. 2004; for a review see Felipo 2006). Previously we showed that the impairment of the NO-cGMP pathway may also result from the interference of Gln with Arg availability for NO synthesis. However, as was suggested by Wu and Meininger (1993) in the study on peripheral endothelial cells, Gln could also affect NO synthesis in the brain by interfering with recycling of the Arg precursor Cit. The capacity of the enzymes involved in Arg regeneration from Cit may represent a rate limiting mechanism in brain cells for maintaining substrate supply for NO synthesis. In relevance to the above consideration, activation of the Cit-NO biosynthetic pathway related to the markedly enhanced ASS, the rate-limiting enzyme in the pathway, was observed in alcoholic liver (Leung et al. 2012). In the present study, ALF caused a parallel increase of ASS activity and iNOS expression. To this point, the present study supports the few previous observations made in other experimental settings. Braissant et al. (1999) showed that expression of ASS and ASL genes is selectively induced in astrocytes treated with 5 mM NH_4_Cl, also suggesting increased recycling of Cit to Arg. Moreover, the study Sharma et al. (2012) on devascularized porcine model of ALF provided indirect evidence for increased de novo synthesis of Arg via Cit-NO cycle.

One as yet unattended step in this cycle of potential rate-limiting impact was the uptake of the Arg precursor Cit. In this study therefore we characterized Cit uptake to the cerebrocortical slices obtained from control rats and rats with TAA-induced ALF, considering this as a potential additive factor in Arg supply for NO synthesis. The study by Albrecht et al. (1996) has analyzed a different stage of the TAA model: the animals were in the recovery period after the TAA insult (7 or 28 days). By contrast rats with TAA induced HE in the model here described (Albrecht et al. 1990) presented increased conversion of Arg to downstream metabolites. There is limited information on Cit transport in brain preparations or cells, and the properties of transporters involved in Cit uptake in peripheral tissues show considerable variations. For example, Cit uptake is predominantly Na^+^ -dependent in rat small interstitium (Vadgama and Evered 1992), but is primarily Na^+^ -independent and mediated by system L, and only partly by system N in rat aortic smooth muscle cells (Wileman et al. 2003). In turn, in pulmonary arterial endothelial cells, exposure to hypoxia evokes system A-mediated Cit uptake (Fike et al. 2012). In rat kidney slices, Cit uptake was largely mediated by the Na^+^-independent organic anion transporter, OAT1 (Nakakariya et al. 2009). The characteristics of Cit uptake to brain slices showed many features in common with those described in aortic smooth muscle cells or rat small interstitium, largely matching transport systems N and L.

Rat cerebral cortical [^3^H]Cit uptake was increased by TAA-induced ALF rat cortex. The increase of V_max_ of the uptake could be partially due to increased activity of the transporter and/or elevated total Cit concentration in the tissue (Table 2); the relative quantitative contribution of either of the two variables remains to be analyzed in more detail. Increased Cit uptake could be ascribed to two transporting systems: N and L. The involvement of the two systems is consistent with a previous report that Cit uptake in a variety of neural cell cultures is Na^+^-independent (Schmidlin et al. 2000). The involvement of the two systems indicates that in the setting of HE, the uptake would show increased sensitivity to, and thus a preponderance to be regulated by, a number of amino acids which are substrates of this system. Of note in this context, system L substrates Gln and Try show increased brain concentration in this model (Hilgier et al. 1992); their specific modulatory role in the Cit-NO cycle remains to be envisaged. While inhibition of Cit uptake by MeAIB and glutamate in TAA cortical slices could suggest the activation of other transport systems (A, x_c_^−^or X_AG_), decreased expression of mRNA expression of two members of system A (SAT1, SAT2) (unpublished observations) tends to exclude this possibility. The involvement of systems: A, x_c_^-^ and X_AG_ in Cit transport deserves further investigation.

In conclusion, increased Cit uptake plus elevation of other parameters of the Cit-NO cycle unambiguously point to its stimulatory effect on NO synthesis in the setting of ALF, as illustrated in Scheme 1. To summarize briefly, Cit concentration in brain homogenates from TAA rat was elevated (Table 2), and so were the activities and expression of the two pertinent enzymes: ASS and ASL (Fig. 4a, b). One other factor with a potential to speed up the operation of the cycle is increased NOS activity. NO synthesis was repeatedly found elevated in different HE models (Schliess et al. 2002; Hilgier et al. 2004; 2009; Sharma et al. 2012), mostly due to increased iNOS, as shown in rats with chronic liver failure due to portacaval shunt (Rodrigo et al. 2007). In agreement with the previously reported data, here we document increased expression of iNOS mRNA in the TAA model of ALF. It remains to be confirmed whether the increase of mRNA is translated to increased iNOS protein level and NOS activity, as suggested by data from other groups (Rodrigo et al. 2007; Balasubramaniyan et al. 2012). Clearly, elucidation the role of NO synthesis in the present model will require an analysis of other NOS isoforms at the level of transcription, translation and enzyme activity. On the other hand, it must be kept in mind that Cit has only a limited ability to sustain maximal rates of NO synthesis in vivo. Exact evaluation of the contribution of Cit transport that could be a regulatory mechanism capable of modulating NO synthesis in the different CNS cell types will require further investigation.

## Abbreviations

ASS: Arginosuccinate synthetase
ASL: Arginosuccinate lyase
NOS: Nitric oxide synthase

## References

[CR1] Albrecht J, Jones EA (1999). Hepatic encephalopathy: molecular mechanisms underlying the clinical syndrome. J Neurol Sci.

[CR2] Albrecht J, Norenberg MD (2006). Glutamine: A Trojan horse in ammonia neurotoxicity. Hepatology.

[CR3] Albrecht J, Hilgier W, Rafałowska U (1990). Activation of arginine metabolism to glutamate in rat brain synaptosomes in thioacetamide-induced hepatic encephalopathy: an adaptative response?. J Neurosci Res.

[CR4] Albrecht J, Hilgier W, Januszewski S, Quack G (1996). Contrasting effects of thioacetamide-induced liver damage on the brain uptake indices of ornithine, arginine and lysine: modulation by treatment with ornithine aspartate. Metab Brain Dis.

[CR5] Balasubramaniyan V, Wright G, Sharma V, Davies NA, Sharifi Y, Habtesion A, Mookerjee RP, Jalan R (2012). Ammonia reduction with ornithine phenylacetate restores brain eNOS activity via the DDAH-ADMA pathway in bile duct-ligated cirrhotic rats. Am J. Physiol Gastrointest Liver Physiol.

[CR6] Bradford MM (1976). A rapid and sensitive method for the quantitation of microgram quantities of protein utilizing the principle of protein-dye binding. Anal Biochem.

[CR7] Braissant O, Honegger P, Loup M, Iwase K, Takiguchi M, Bachmann C (1999) Hyperammonemia: regulation of argininosuccinate synthetase and argininosuccinate lyase genes in aggregating cell cultures of fetal rat brain. Neurosci Lett 266:89–92 10.1016/s0304-3940(99)00274-8 10353334

[CR8] Cooper AJ, Plum F (1987). Biochemistry and physiology of brain ammonia. Physiol Rev.

[CR9] Felipo V (2006). Contribution of altered signal transduction associated to glutamate receptors in brain to the neurological alterations of hepatic encephalopathy. World J Gastroenterol.

[CR10] Felipo V, Butterworth RF (2002). Neurobiology of ammonia. Prog Neurobiol.

[CR11] Fike CD, Sidoryk-Wegrzynowicz M, Aschner M, Summar M, Prince LS, Cunningham G, Kaplowitz M, Zhang Y, Aschner JL (2012). Prolonged hypoxia augments L-citrulline transport by system A in the newborn piglet pulmonary circulation. Cardiovasc Res.

[CR12] Fotiadis D, Kanai Y, Palacín M (2013). The SLC3 and SLC7 families of amino acid transporters. Molecular Aspects of Medicine.

[CR13] Garthwaite J, Charles SL, Chess Williams R (1988). Endothelium-derived relaxing factor release on activation of NMDA receptors suggests role as intercellular messenger in the brain. Nature.

[CR14] Hazell AS, Norenberg MD (1998). Ammonia and manganese increase arginine uptake in cultured astrocytes. Neurochem Res.

[CR15] Hermenegildo C, Montoliu C, Llansola M, Munoz MD, Gaztelu JM, Minana MD, Felipo V (1998). Chronic hyperammonemia impairs the glutamate-nitric oxide-cyclic GMP pathway in cerebellar neurons in culture and in the rat in vivo. Eur J Neurosci.

[CR16] Hermenegildo C, Monfort P, Felipo V (2000). Activation of N-methyl-D-aspartate receptors in rat brain in vivo following acute ammonia intoxication: characterization by in vivo brain microdialysis. Hepatology.

[CR17] Hilgier W, Olson JE (1994). Brain ion and amino acid contents during edema development in hepatic encephalopathy. J Neurochem.

[CR18] Hilgier W, Puka M, Albrecht J (1992). Characteristics of large neutral amino acid-induced release of preloaded L-glutamine from rat cerebral capillaries in vitro: effects of ammonia, hepatic encephalopathy, and gamma-glutamyl transpeptidase inhibitors. J Neurosci Res.

[CR19] Hilgier W, Olson JE, Albrecht J (1996). Relation of taurine transport and brain edema in rats with simple hyperammonemia or liver failure. J Neurosci Res.

[CR20] Hilgier W, Oja SS, Saransaari P, Albrecht J (2004). A novel glycine site-specific N-methyl-D-aspartate receptor antagonist prevents activation of the NMDA/NO/CGMP pathway by ammonia. Brain Res.

[CR21] Hilgier W, Fresko I, Klemenska E, Beresewicz A, Oja SS, Saransaari P, Albrecht J, Zielinska M (2009). Glutamine inhibits ammonia-induced accumulation of cGMP in rat striatum limiting arginine supply for NO synthesis. Neurobiol Dis.

[CR22] Ishihara T, Takada T, Shoji Y, Uedono Y, Takeyama N, Tanaka T (1998). Hyperammonemia reduces water immersion–restraint stress gastric ulcers in rats. Gen Pharmacol.

[CR23] Kosenko E, Llansola M, Montoliu C, Monfort P, Rodrigo R, Hernandez-Viadel M, Erceg S, Sánchez-Perez AM, Felipo V (2003). Glutamine synthetase activity and glutamine content in brain: modulation by NMDA receptors and nitric oxide. Neurochem Int.

[CR24] Leung TM, Lu Y, Yan W, Morón-Concepción JA, Ward SC, Ge X, Conde de la Rosa L, Nieto N (2012). Argininosuccinate synthase conditions the response to acute and chronic ethanol-induced liver injury in mice. Hepatology.

[CR25] Livak KJ, Schmittgen TD (2001). Analysis of relative gene expression data using real-time quantitative PCR and the 2(-Delta Delta C(T)) method. Methods.

[CR26] Martinez-Hernandez A, Bell KP, Norenberg MD (1977). Glutamine synthetase: glial localization in brain. Science.

[CR27] Nakakariya M, Shima Y, Shirasaka Y, Mitsuoka K, Nakanishi T, Tamai I (2009). Organic anion transporter OAT1 is involved in renal handling of citrulline. Am J Physiol Renal Physiol.

[CR28] Prakash R, Mullen KD (2010). Mechanisms, diagnosis and management of hepatic encephalopathy. Nat Rev Gastroenterol Hepatol.

[CR29] Rao VL (2002). Nitric oxide in hepatic encephalopathy and hyperammonemia. Neurochem Int.

[CR30] Rao VL, Butterworth RF (1996). L-[3H]Nitroarginine and L-[3H]arginine uptake into rat cerebellar synaptosomes: kinetics and pharmacology. J Neurochem.

[CR31] Rao VL, Audet RM, Butterworth RF (1997). Portacaval shunting and hyperammonemia stimulate the uptake of L-[3H] arginine but not of L- [3H]nitroarginine into rat brain synaptosomes. J Neurochem.

[CR32] Rodrigo R, Erceg S, Rodriguez-Diaz J, Saez-Valero J, Piedrafita B, Suarez I, Felipo V (2007) Glutamate-induced activation of nitric oxide synthase is impaired in cerebral cortex in vivo in rats with chronic liver failure. J Neurochem 102:51–64 10.1111/j.1471-4159.2006.04446.x 17286583

[CR33] Schliess F, Görg B, Fischer R, Desjardins P, Bidmon HJ, Herrmann A, Butterworth RF, Zilles K, Häussinger D (2002). Ammonia induces MK-801-sensitive nitration and phosphorylation of protein tyrosine residues in rat astrocytes. FASEB J.

[CR34] Schmidlin A, Fischer S, Wiesinger H (2000). Transport of L-citrulline in neural cell cultures. Dev Neurosci.

[CR35] Sharma V, Ten Have GA, Ytrebo L, Sen S, Rose CF, Dalton RN, Turner C, Revhaug A, van-Eijk HM, Deutz NE, Jalan R, Mookerjee RP, Davies NA (2012) Nitric oxide and L-arginine metabolism in a devascularized porcine model of acute liver failure. Am J Physiol Gastrointest Liver Physiol 303:G435–441 10.1152/ajpgi.00268.2011 PMC3774247 22421619

[CR36] Swamy M, Zakaria AZ, Govindasamy C, Sirajudeen KN, Nadiger HA (2005). Effects of acute ammonia toxicity on nitric oxide (NO), citrulline-NO cycle enzymes, arginase and related metabolites in different regions of rat brain. Neurosci Res.

[CR37] Vadgama JV, Evered DF (1992). Characteristics of L-citrulline transport across rat small intestine in vitro. Pediatr Res.

[CR38] Westergaard N, Beart PM, Schousboe A (1993). Transport of L-[3H]arginine in cultured neurons: characteristics and inhibition by nitric oxide synthase inhibitors. J Neurochem.

[CR39] Wileman SM, Mann GE, Pearson JD, Baydoun AR (2003). Role of L-citrulline transport in nitric oxide synthesis in rat aortic smooth muscle cells activated with LPS and interferon-g. Br J Pharmacol.

[CR40] Wu G, Meininger CJ (1993). Regulation of L-arginine synthesis from L-citrulline by L-glutamine in endothelial cells. Am J Physiol.

[CR41] Zhang WY, Takiguchi M, Koshiyama Y, Gotoh T, Nagasaki A, Iwase K, Yamamoto K, Takshima H, Negi A, Mori M (1999). Expression of citrulline–nitric oxide cycle in lipopolysaccharide and cytokine-stimulated rat astroglioma C6 cells. Brain Res.

[CR42] Zielińska M, Hilgier W, Law RO, Goryński P, Albrecht J (1999). Effects of ammonia in vitro on endogenous taurine efflux and cell volume in rat cerebrocortical minislices: influence of inhibitors of volume-sensitive amino acid transport. Neuroscience.

[CR43] Zielinska M, Ruszkiewicz J, Hilgier W, Fresko I, Albrecht J (2011). Hyperammonemia increases the expression and activity of the glutamine/arginine transporter y + LAT2 in rat cerebral cortex: implications for the nitric oxide/cGMP pathway. Neurochem Int.

